# Spider Bite: A Rare Case of Acute Necrotic Arachnidism with Rapid and Fatal Evolution

**DOI:** 10.1155/2016/7640789

**Published:** 2016-08-29

**Authors:** Mario Pezzi, Anna Maria Giglio, Annamaria Scozzafava, Orazio Filippelli, Giuseppe Serafino, Mario Verre

**Affiliations:** Anaesthesia and Intensive Care Unit, General Hospital “Pugliese-Ciaccio”, Viale Pio X, 88100 Catanzaro, Italy

## Abstract

The spider bites are quite frequent and often resolve quickly without leaving outcomes; only some species are capable of causing necrotic and systematic lesions in humans. Among them, we should mention the genus* Loxosceles*. The venom released from the spider bite of* Loxosceles* species is composed of proteins, enzymes, and nonenzymatic polypeptides. The phospholipase D family was identified as the active component of the venom. This family of enzymes is responsible for the local and systemic effects observed in loxoscelism. Phospholipases D interact with cell membranes triggering alterations which involve the complement system and activation of neutrophils and they cause the dermonecrotic skin lesions and systemic effects. We describe a fatal case of acute intoxication caused by a spider bite probably belonging to the species* Loxosceles*. The initial lesion was localized to a finger of a hand. Clinical course was worsening with deep necrotic lesions on limb, shock, hemolysis, acute kidney failure, and disseminated intravascular coagulation. All therapies were ineffective. This is the first fatal case described in Europe.

## 1. Introduction

The spider bites are quite frequent and often resolve quickly without leaving outcomes; only some species are capable of causing necrotic and systematic lesions in humans; among them, we should mention the genus* Loxosceles*. The common morphological features of these spiders are three pairs of eyes (instead of eight), the particular dark spot on the back often violin-shaped, and frequently brown in colour of different shades.

The species* Loxosceles rufescens* is widespread in the Mediterranean area [1] and thus extends throughout the Italian territory (including Calabria region). It belongs to the Sicariidae family (from the Latin word* sicarium* meaning murderess, for the toxicity of its venom), Araneae order, and Arachnida class. It is a sedentary spider with nocturnal activity and is often found in dark and little frequented places such as basements, attics, ceilings, and garages. The venom is composed of low molecular weight proteins, proteolytic enzymes, and nonenzymatic polypeptides with a hemolytic-necrotic action (hence the term necrotic arachnidism) causing oedema, necrosis, and deep ulcerations of the affected parts (skin loxoscelism). Frequently at the bite site, after 12–24 hours, a characteristic necrotic lesion forms circled by a bluish area and a whitish ring (shaped like a bull's eye), which is very painful. It is hard to correlate the severity of the clinical outlook with the individual components of the venom, but, certainly, these are complex molecular mechanisms which involve the host's response [2]. It has been demonstrated that the venom of the* Loxosceles* genus is a potent inducer of the inflammatory response mediated by cytokines and lymphocytes [3].

In rare cases, especially for the* Loxosceles rufescens*, skin necrosis can spread into subcutaneous tissue and underlying muscles developing a systematic toxicity with varying degrees of severity. Mild-moderate systemic effects involve fever, malaise, myalgia, arthralgia, and rash. In severe but fortunately rare forms, 24–48 hours from the bite, hemolysis, rhabdomyolysis, jaundice, acute renal insufficiency up to shock, and disseminated intravascular coagulation can be observed. This is the case of a patient with a rare form of systemic necrotic arachnidism likely caused by a* Loxosceles rufescens* bite with rapid development.

## 2. Clinical Case

A woman of 65, obese (BMI = 44.06), with no history of diabetes and allergies but with a mild form of myasthenia gravis (treated only with pyridostigmine po 60 mg every 6 hours), was bitten the evening before hospitalization while cleaning the home cellar by a spider, which, from the description and place where the bite occurred, could probably be identified as the* Loxosceles rufescens* species. It was not possible to capture the spider. It was also not possible to visit the home cellar, but we had confirmation from local health service of a* Loxosceles* infestation in neighboring houses.

Initially, the patient did not give much thought to the event given the few or no symptoms but, after the night, early in the morning, given the sharp pain in her right hand, where a bullous lesion had appeared in the middle phalanx of the third finger, accompanied by malaise and fever (38.2°C), she was admitted to a nearby emergency room.

In our Intensive Care Unit, the patient arrived after about four hours owing to the progressive worsening of her general clinical condition. To our observation, after noninvasive monitoring of vital signs, she appeared drowsy (GCS = 8) and tachypneic (respiratory rate = 28/min), with heart rate 90/min and blood pressure = 82/55 mmHg. We could see a circular necrotic skin lesion on the middle phalanx of the third finger of her right hand with erythroderma and oedema of the hand which partially affected the forearm, with strong pain symptoms.

The limb arterial and venous circulation seemed to have stalled, after we performed an ultrasound examination. Telematic support from the National Poison Control Centre in Milan (Italy) confirmed acute necrotic arachnidism from the information provided.

A bolus of saline 30 mL/kg IV, morphine 5 mg IV, and dexamethasone 4 mg were administered to the patient and high flow oxygen via nasal cannula (HFNC) was initiated. Blood tests revealed the following abnormalities: white blood cells 3.3 *∗* 10^3^ *μ*L, Hb 4.59 mmol/L, platelets 55.000 *∗* 10^3^ *μ*L, INR 1.72, PT 38.2%, aPTT 121 sec., Procalcitonin 92 ng/mL, and glucose level of 10.67 mmol/L. The blood gas analysis showed a severe metabolic acidosis with pH 7.12, lactate 8.4 mmol/L, and HCO_3_ 10 mmol/L.

Endotracheal intubation was initiated along with mechanical ventilation in controlled pressure and invasive monitoring of blood pressure and central venous pressure by placement of a central venous catheter with double lumen 14 Fr catheter. Treatment with norepinephrine at 0.15 mcg/kg/min was started as well as dopamine 8 mcg/kg/min; sedation was achieved via ongoing infusion of remifentanil and midazolam. Prophylaxis against tetanus, specific immunoglobulins, and toxoid tetanus were administered to her. A blood and wound pad culture were taken (which turned out negative after) and broad-spectrum antibiotic coverage was started with Meropenem with 1 gr × 3 doses iv in 24 hours and infusion iv of Daptomycin 4 mg/kg in 30 minutes.

**Figure 1:**
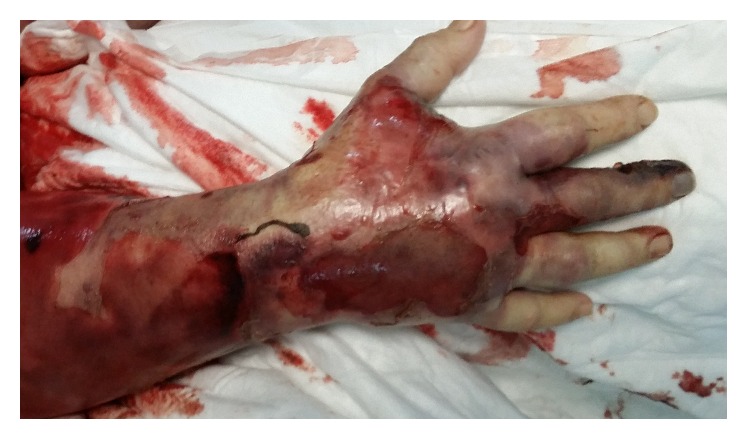
Tissue injury with appearance of erythroderma bullosa.

**Figure 2:**
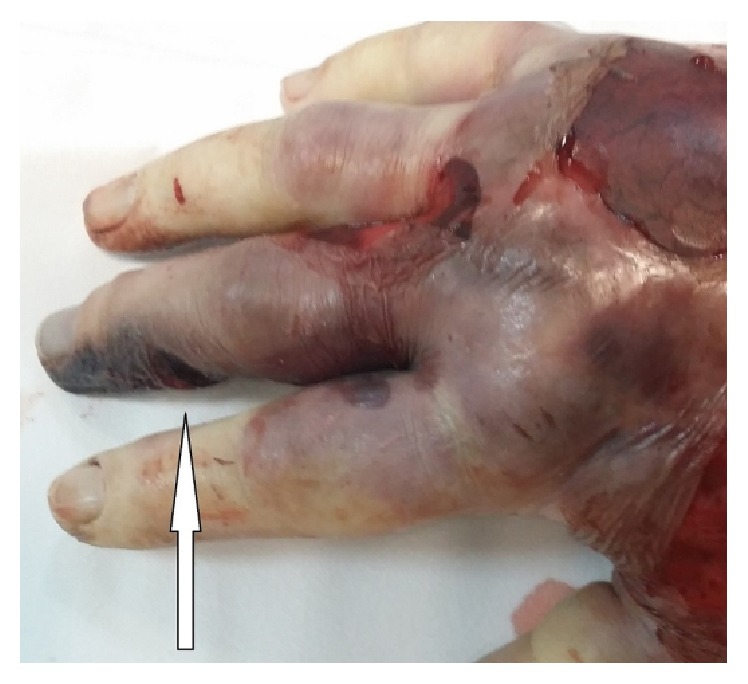
Initial necrotic lesion (white arrow).

**Figure 3:**
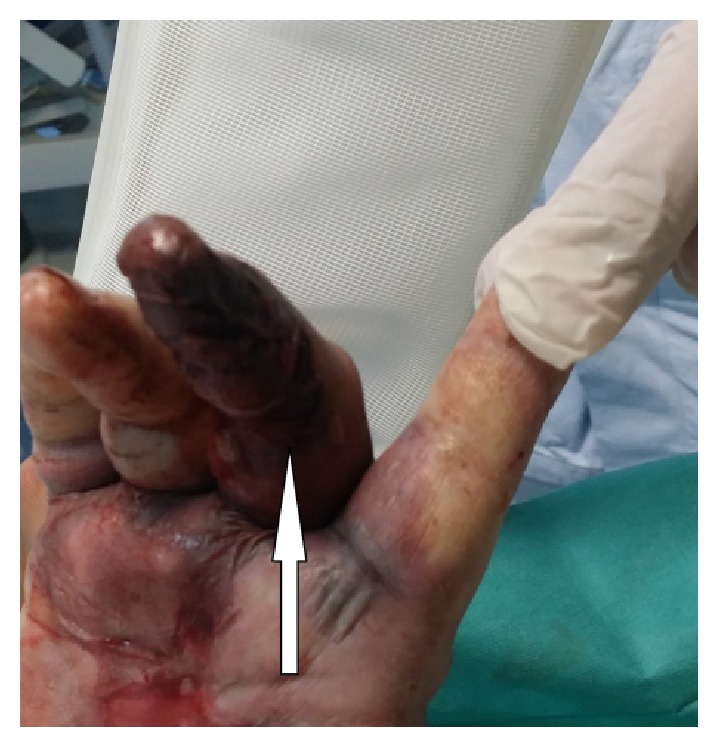
Initial necrotic lesion (white arrow).

About six hours from admission to the ICU, the oedema had spread to part of the arm with the appearance of erythroderma bullosa (Figure 1) and was clearly distinguished from the initial necrotic lesion on the middle phalanx (Figures 2 and 3). Owing to the development of severe rhabdomyolysis (creatine kinase 2994 U/L and myoglobin 2000 ng/mL), the patient underwent CPFA (coupled plasma filtration adsorption). Transfusion of concentrated red cells was performed due to progressive hemolytic anaemia. Diuresis was kept at values of 1.2 mL/kg/min.

**Figure 4:**
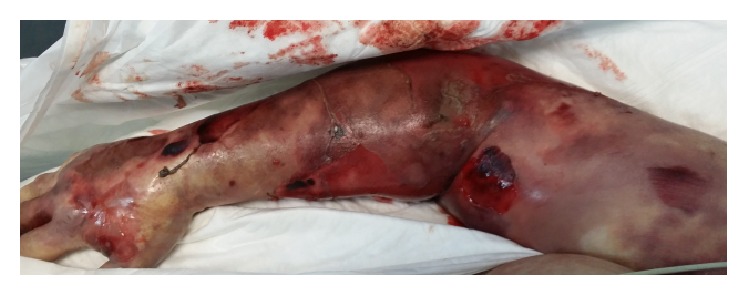
Deterioration and extension of necrotic tissue injury.

About 12 hours from admission, there was a worsening in tissue condition (Figure 4), appearance of refractory shock, and disseminated intravascular coagulation leading to death of the patient.

## 3. Discussion

There are four categories of* Loxosceles* bites [4]:

- Unremarkable (very little damage and self-healing)
- Mild (redness, itching, and slight lesion but typically self-healing)
- Dermonecrotic (necrotic skin lesion considered by many as the typical reaction)
- Systemic or viscerocutaneous (affecting vascular system, very rare, and potentially fatal)

The venom of* Loxosceles* species is composed of a number of proteins, enzymes, and nonenzymatic polypeptides. Phospholipases D are identified as deleterious components of venom involved in noxious activities. This family of enzymes is described as producing choline to generate ceramide-1-phosphate from sphingomyelin (SM), or lysophosphatidic acid from lysophosphatidylcholine, profoundly altering the lateral structure and morphology of the target membrane, since lysophosphatidylcholine also is a substrate for enzymes found in cytoplasmic cell membranes [5].

Phospholipases D interact with cell membranes and other elements in tissue triggering alterations which involve the complement system and activation of neutrophils. More important is the response of host cells to the presence of a complement mediating foreign substance [6].

Painful bites may be noticed immediately and the offending spider may be seen; often, a diagnosis is made in retrospect from a characteristic history and physical examination.

It is now well established that phospholipase D family is the cause for dermonecrotic skin lesions in humans bitten by* Loxosceles* arachnids and is the main component of the venom responsible for the local and systemic effects observed in loxoscelism.* Loxosceles* venom is a potent inducer of multiple inflammatory mediating chemokines such as IL-8 and chemokine growth-regulated protein alpha (GRO-alpha) or chemokines including monocyte chemoattractant protein-1 (MCP-1) [7].

In severe cases, cutaneous necrosis may occur and may extend to involve subcutaneous fat and muscle. Systemic effects may develop, usually 24 to 72 hours after bite. Severe systemic effects include fever, chills, myalgia, arthralgia and generalized rash, hemolysis, jaundice, rhabdomyolysis, renal failure, disseminated intravascular coagulation, coma, and shock [8]. In this case, the simultaneous presence of myasthenia gravis, an antibody mediated disease, may have perhaps favored the fulminating evolution of symptoms. Patients with evidence of hemolysis, significant infection, severe systemic effects, or complicated wounds require hospital admission. Various types of treatments have been described for the management of cutaneous and visceral loxoscelism: corticosteroids, dapsone, colchicine, hyperbaric oxygen, blood transfusions, antimicrobials, surgical treatment, vasodilators, antihistamines, anticoagulants, and serum anti-*Loxosceles* (South America) [9]. Treatment for systemic effects includes support of respiratory and cardiovascular function and hydration [10]. Transfusion may be necessary in patients with severe hemolysis.

Success of therapy depends upon a correct and rapid diagnosis, the volume of the venom injected, and the patient susceptibility to the venom [11].

## 4. Conclusions

The deaths from spider bites are very rare in the world [12].

In the Mediterranean area, the poisonings by* Loxosceles rufescens* spider bite are rarely described in the medical literature [13].

So far, no case of death was described due to the bite of* Loxosceles rufescens* in Europe.

The clinical case described by us was very serious, rapidly progressive, and refractory to any therapy.
